# Why not try to predict autism spectrum disorder with crucial biomarkers in cuproptosis signaling pathway?

**DOI:** 10.3389/fpsyt.2022.1037503

**Published:** 2022-11-02

**Authors:** Yu Zhou, Jing Gao

**Affiliations:** ^1^Department of Child Rehabilitation Division, Huai’an Maternal and Child Health Care Center, Huai’an, China; ^2^Affiliated Hospital of Yang Zhou University Medical College, Huai’an Maternal and Child Health Care Center, Huai’an, China

**Keywords:** cuproptosis, autism spectrum disorder, biomarkers, machine learning, artificial neural network

## Abstract

The exact pathogenesis of autism spectrum disorder (ASD) is still unclear, yet some potential mechanisms may not have been evaluated before. Cuproptosis is a novel form of regulated cell death reported this year, and no study has reported the relationship between ASD and cuproptosis. This study aimed to identify ASD in suspected patients early using machine learning models based on biomarkers of the cuproptosis pathway. We collected gene expression profiles from brain samples from ASD model mice and blood samples from humans with ASD, selected crucial genes in the cuproptosis signaling pathway, and then analysed these genes with different machine learning models. The accuracy, sensitivity, specificity, and areas under the receiver operating characteristic curves of the machine learning models were estimated in the training, internal validation, and external validation cohorts. Differences between models were determined with Bonferroni’s test. The results of screening with the Boruta algorithm showed that FDX1, DLAT, LIAS, and ATP7B were crucial genes in the cuproptosis signaling pathway for ASD. All selected genes and corresponding proteins were also expressed in the human brain. The k-nearest neighbor, support vector machine and random forest models could identify approximately 72% of patients with ASD. The artificial neural network (ANN) model was the most suitable for the present data because the accuracy, sensitivity, and specificity were 0.90, 1.00, and 0.80, respectively, in the external validation cohort. Thus, we first report the prediction of ASD in suspected patients with machine learning methods based on crucial biomarkers in the cuproptosis signaling pathway, and these findings may contribute to investigations of the potential pathogenesis and early identification of ASD.

## Introduction

Autism spectrum disorder (ASD) is defined as a group of neurodevelopmental psychiatric disorders characterized by deficits in social interactions, interpersonal communications, and repetitive and stereotyped behaviors and can accompany other disorders, such as intellectual and language disorders (1). Although ASD can be diagnosed as early as 18–24 months of age, a significant proportion of children are not identified until the school years (2, 3). Early identification of ASD in children could improve developmental outcomes and quality of life through early intervention.

The genetic influence of autism is complex and possibly related to environmental factors (4). ASD has been found to be associated with many physiological abnormalities, including reactive oxygen species (ROS), mitochondrial dysfunction, intracellular calcium ion level regulation and even the gut microbiota (5–7). However, there is no established biomarker for ASD diagnosis. Thus, in the past, some physiological processes and biomarkers for ASD and diagnosis may have been ignored.

A recent study published in *Science* by Tsvetkov et al. showed that intracellular copper (Cu) induced a novel form of cell death (8), named cuproptosis. Cuproptosis is mainly regulated by ferredoxin 1 (FDX1)-mediated mitochondrial proteotoxic stress. The authors indicated that FDX1 could reduce Cu^2+^ to Cu^+^ and promote the lipoylation and aberrant oligomerization of DLAT, which is involved in the regulation of the mitochondrial tricarboxylic acid cycle. Glutathione (GSH) blocks cuproptosis by chelating intracellular Cu. In addition, lipoic acid synthetase (LIAS) decreases cell sensitivity to cuproptosis by blocking the lipoylation of proteins. Solute carrier family 31 member 1 (SLC31A1) and ATPase copper transporting beta (ATP7B) affect cuproptosis sensitivity by regulating the level of intracellular Cu^+^. However, no study has revealed the relationship between ASD and crucial genes for cuproptosis thus far.

Predicting the incidence of disease has been a challenging task in the past. In recent years, the development of machine learning methods has allowed us to envision a future of improved health care through the investigation of biomedical profiles and patient datasets (9). A recent study showed that the use of machine learning methods in Alzheimer’s disease shows promise for the identification of novel molecular characterizations (10), while those methods are not still being investigated in ASD.

Hence, we aimed to investigate some novel biomarkers in the cuproptosis signaling pathway for ASD through the use of machine learning algorithms. To support our goals, we collected gene expression profiles from brain tissue samples from ASD model mice and peripheral blood samples from humans with ASD. Then, we selected crucial genes in the cuproptosis signaling pathway for ASD and verified these features with different machine learning algorithms.

## Materials and methods

### Data collection

The gene expression data of ASD mouse brain samples were obtained from the Gene Expression Omnibus (GEO) database (GSE72149 and GSE81501). The gene expression data of peripheral blood samples from 20 children with ASD and 20 healthy control children were also obtained (GSE26415). All genes in the expression profiles were annotated as unique gene symbols, and expression values were transformed by log2. Then, expression values were normalized with the “limma” package in R software to achieve consistency and comparability between arrays. The differentially expressed genes (DEGs) were screened by the “limma” package according to a previous study (11). If the *p*-value was < 0.01 between arrays, the corresponding gene was considered a DEG.

### Visualization of crucial genes in the cuproptosis signaling pathway

We selected six crucial genes of cuproptosis regulation reported as candidate biomarkers in a previous study, including FDX1, DLAT, LIAS, GSH, ATP7B, and SLC31A1 (8). Selected genes were visualized in a heatmap created with the “pheatmap” package in R software.

### Screened risk factor genes in the cuproptosis signaling pathway

The FDX1, DLAT, LIAS, GSH, ATP7B, and SLC31A1 expression data were evaluated by the Boruta algorithm. The Boruta method, which has shown reasonable reliability for feature selection in many fields, and is considered one of the most powerful algorithms for analyzing large data sets (12–14). This method was built around the random forest classifier to determine the relevance and importance of in relation to the target variables (15). Thus, we used the Boruta algorithm to select risk features in the present study.

We next divided the gene expression data of ASD mice into a training cohort (70%) and an internal validation cohort (30%), and the peripheral blood gene expression profiles of humans with ASD were used as an external validation cohort.

### Expression of selected genes and subcellular localization in the human brain

All selected risk genes in the cuproptosis signaling pathway were detected in the Human Protein Atlas database (Version: 21.1).^1^ This database maps all human proteins in cells, tissues, and organs using an integration of various omics technologies, including antibody-based imaging, mass spectrometry-based proteomics, transcriptomics, and systems biology (16). This database has been used in many studies (17–19). The expression levels of four selected genes were measured in different parts of the human brain, and protein expression analysis was used to determine the locations of protein expression in cells.

### Verification with different machine learning methods

The risk factor genes in the cuproptosis signaling pathway screened with the Boruta algorithm were verified by five frequently used machine learning methods, including k-nearest neighbor (KNN), naive Bayesian (NB), support vector machine (SVM) with polynomial kernel, random forest (RF), and artificial neural network (ANN). All five machine learning models were trained in the training cohort and verified in the internal validation cohort and external validation cohort.

k-nearest neighbor performs classification by assigning a point to the class that is most prevalent out of the k points closest to it (20). The k parameter was set between 2 and 20 in the present study, and the optimized k value was chosen (usually an odd number). KNN was performed with the “kknn” package in R.

Naive Bayesian is conducted based on Bayes’ theorem and finds the probability that an input with some features belongs to a certain class (21). NB was conducted by the “e1071” package in R software.

Support vector machine performs input data as feature vectors and calculates them in a space with the same dimensionality, divides the data points into two categories, and finally selects the optimal hyperplane (22). SVM was performed by the “e1071” package in R software.

Random forest is made up of decision trees with slight differences. RF can classify input data into the most common classifications based on constituent decision trees (23). The optimized number of trees was selected for the next validation, and RF was pruned to combat their tendency to overfit. RF was conducted by the “randomForest” package.

The ANN was made up of several layers of neurons and could loosely mimic the learning method in human brains (24). The number of hidden layers was set to five to six in the present study, and the sigmoid function was used as the standard activation method. ANN was performed with the “neuralnet” package in R software.

### Statistical analysis

The true condition was set to ASD or control in different cohorts. The prediction accuracy and its 95% confidence interval (CI) and kappa statistic values were calculated in the training, internal validation, and external validation cohorts for all models. For repeatability, a fixed seed number was set before cross validation. Receiver operating characteristic (ROC) curves were plotted for the internal validation cohort and external validation cohort, and the area under the curve (AUC) was calculated to examine the performance of different machine learning models.

The “resamples” package in R was used to analyse and visualize the performance of each model after cross validation. Differences between paired machine learning methods were determined with Bonferroni’s test (25).

## Results

### Data normalization and visualization

**FIGURE 1:**
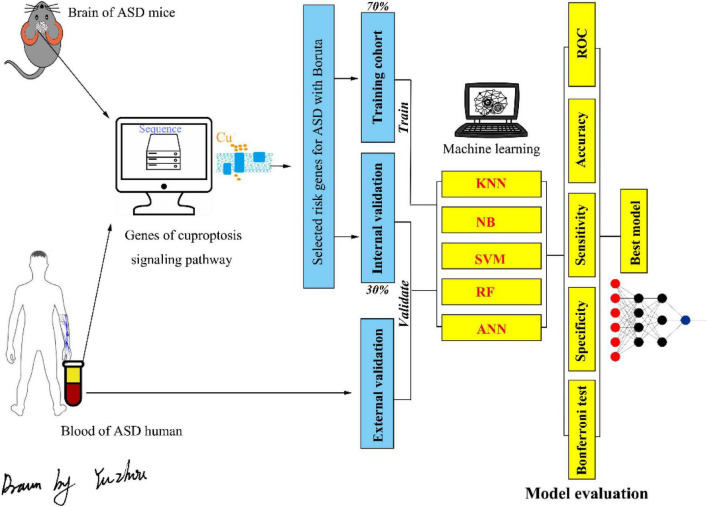
The flowchart of the present study. ASD, autism spectrum disorder; KNN, k-nearest neighbor; NB, naive Bayesian; SVM, support vector machine; RF, random forest; ANN, artificial neural network.

**FIGURE 2:**
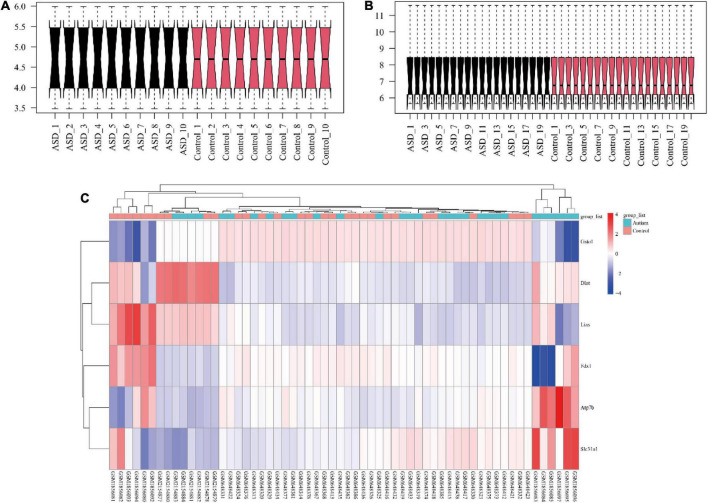
The visualization of gene expression profiles. The gene expression data were normalized between arrays of mouse brain **(A)** and human blood **(B)**. The crucial genes in the cuproptosis signaling pathway and the selected arrays of each dataset were visualized in a heatmap **(C)**. ASD, autism spectrum disorder.

Twenty brain expression datasets of ASD and control mice (10 each) were collected from GSE72149 and GSE81501. Twenty peripheral blood gene expression profiles of children with ASD and 20 age- and sex-matched peripheral blood gene expression profiles of healthy controls were collected from GSE26415. The flowchart of the data analysis is shown in Figure 1. As shown in Figure 2, the expression data were normalized between the arrays in each dataset.

### Visualization of crucial genes in the cuproptosis signaling pathway and the selection of risk features for autism spectrum disorder

The selected expression arrays in each cohort and crucial genes in the cuproptosis signaling pathway were visualized with a heatmap (Figure 2C). The results of Boruta analysis showed that FDX1, DLAT, LIAS, and ATP7B were identified as feature genes, and other genes were classified as unimportant feature genes in the present data.

### Expression of selected genes and the location of proteins in the human brain

**FIGURE 3:**
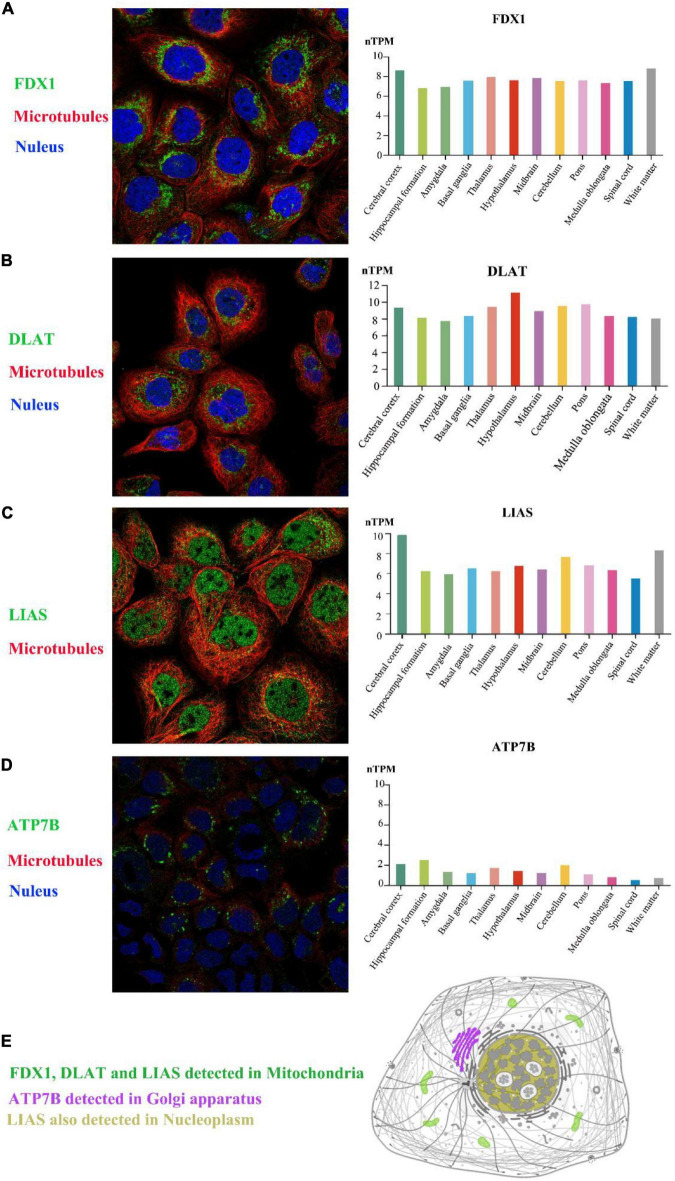
The expression of selected genes in the brain. Based on the Human Protein Atlas, FDX1, DLAT, LIAS, and ATP7B could all be detected in 12 brain regions. In the A-431 cell line, FDX1 protein and DLAT protein were located in mitochondria **(A,B)**, and LIAS protein was located in mitochondria and the nucleoplasm **(C)**. ATP7B protein was also expressed in the Golgi apparatus in the CACO-2 cell line **(D)**. The schematic graph shows the main location of each protein in cells **(E)**. The target proteins, nuclei and microtubules were stained green, blue, and red, respectively. nTPM, normalized transcript expression values.

The expression profiles of humans with ASD were obtained from blood; however, whether these risk genes are expressed in the human brain is still unclear. Based on Human Protein Atlas immunofluorescence analysis, FDX1, and DLAT were located in mitochondria, ATP7B was expressed in the Golgi apparatus, and LIAS could be detected in mitochondria and the nucleoplasm (Figure 3). In addition, the four selected genes were all expressed in the main parts of the brain. Thus, these four genes could be detected in the brains of mice and both the blood and the brains of humans.

### Modeling by k-nearest neighbor

**FIGURE 4:**
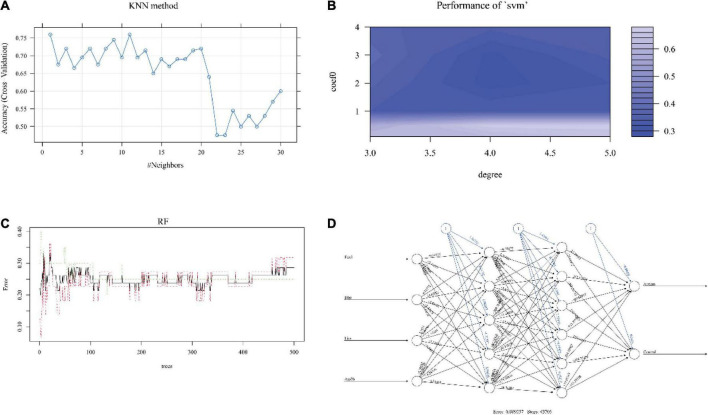
The performance of each machine learning model. The relation between the number of neighbors (k value) and accuracy in KNN **(A)**. The degree and coefficient of SVM are shown in panel **(B)**. In RF, the interrelation between the number of trees and model error is shown in panel **(C)**. After 43,703 steps, the error was 0.009257 in the ANN model **(D)**. KNN, k-nearest neighbor; SVM, support vector machine; RF, random forest; ANN, artificial neural network.

**FIGURE 5:**
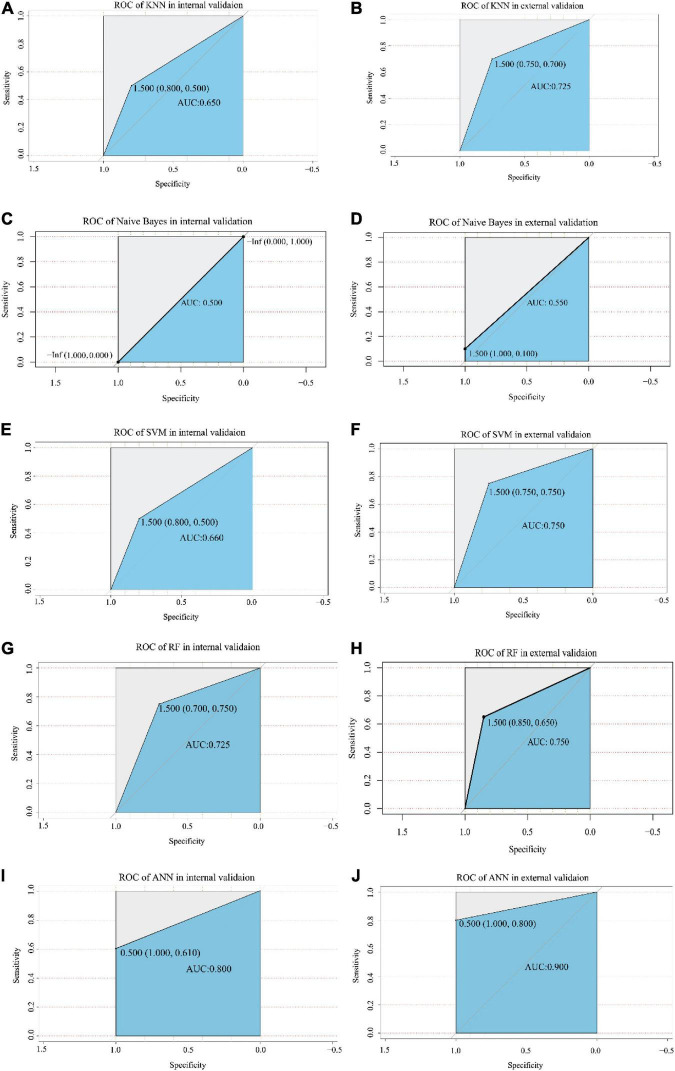
The ROC analysis of each model in the internal validation and external validation cohorts. The X-axis and Y-axis represent specificity and sensitivity, respectively. The AUC values are indicated in the blue area, including KNN model **(A,B)**, Naive Bayes model **(C,D)**, SVM model **(E,F)**, RF model **(G,H)**, and ANN model **(I,J)**. The value of the cut-off point is shown at the inflection point. ROC, receiver operating characteristic; AUC, area under the curve; KNN, k-nearest neighbor; SVM, support vector machine; RF, random forest; ANN, artificial neural network.

The optimized k value was set as 11 (Figure 4A). In the training cohort, the accuracy was 0.76 (95% CI, 0.60–0.88), and the sensitivity and specificity were 0.80 and 0.72, respectively. In the internal validation cohort, the accuracy was 0.67 (95% CI, 0.51–0.87); the sensitivity and specificity were 0.80 and 0.50, respectively; and the AUC was 0.650 (Table 1 and Figure 5A). The accuracy, sensitivity, and specificity were 0.73 (95% CI, 0.56–0.86), 0.75 and 0.70, respectively (Table 1), and the AUC was 0.725 in the external validation cohort (Figure 5B).

**TABLE 1:** Accuracy, sensitivity, and specificity of each machine learning model.

Model types	Cohorts	Accuracy (95% CI)	Sensitivity	Specificity
KNN	Training cohort	0.76 (0.60–0.88)	0.80	0.72
	Internal validation	0.67 (0.51–0.87)	0.80	0.50
	External validation	0.73 (0.56–0.86)	0.75	0.70
NB	Training cohort	0.64 (0.50–0.78)	0.95	0.36
	Internal validation	0.56 (0.41–0.78)	1.00	0.00
	External validation	0.55 (0.40–0.70)	1.00	0.10
SVM	Training cohort	0.89 (0.74–0.96)	0.85	0.91
	Internal validation	0.68 (0.42–0.87)	0.80	0.51
	External validation	0.75 (0.59–0.87)	0.75	0.75
RF	Training cohort	0.83 (0.69–0.93)	0.95	0.73
	Internal validation	0.72 (0.52–0.90)	0.70	0.75
	External validation	0.75 (0.59–0.87)	0.85	0.65
ANN	Training cohort	1.00 (0.92–1.00)	1.00	1.00
	Internal validation	0.78 (0.62–0.94)	1.00	0.61
	External validation	0.90 (0.76–0.97)	1.00	0.80

### Modeling by naive Bayesian

The results showed that the accuracy of NB was 0.64 (95% CI, 0.50–0.78), the sensitivity was 0.95, and the specificity was 0.36 in the training dataset. The accuracy was 0.56 (95% CI, 0.41–0.78) and 0.55 (95% CI, 0.40–0.70) in the internal validation cohort and external validation cohort, respectively. The sensitivity of the internal validation cohort and external validation cohort was 1.00, but the specificity was zero in the internal validation cohort and only 0.1 in the external validation cohort (Table 1). The AUC values were 0.500 and 0.550 in the internal validation cohort and external validation cohort, respectively (Figures 4D, 5C).

### Modeling by support vector machine

The number of support vectors was 25 with the best SVM model in the present study, and the performance of the SVM is shown in Figure 4B. With the best SVM model, accuracy, sensitivity, and specificity were 0.89 (95% CI, 0.74–0.96), 0.85 and 0.91, respectively, in the training cohort (Table 1). In the internal validation cohort, the accuracy, sensitivity, specificity and AUC were 0.68 (95% CI, 0.42–0.87), 0.80, 0.51, and 0.660, respectively (Table 1 and Figure 5E). The accuracy, sensitivity, specificity, and AUC were 0.75 (95% CI, 0.59–0.87), 0.75, 0.75, and 0.750, respectively, in the external validation cohort (Table 1 and Figure 5F).

### Modeling by random forest

The RF was performed with an optimized tree number (Figure 4C). The accuracy, sensitivity and specificity in the training dataset with RF were 0.83 (95% CI, 0.69–0.93), 0.95, and 0.73, respectively. The accuracy, sensitivity, specificity and AUC were 0.72 (95% CI, 0.52–0.90), 0.70, 0.75, and 0.725 in the internal validation cohort, respectively (Table 1 and Figure 5G). In the external validation cohort, the accuracy, sensitivity, specificity and AUC were 0.75 (95% CI, 0.59–0.87), 0.85, 0.65, and 0.750, respectively (Table 1 and Figure 5H).

### Modeling by artificial neural network

We first trained the ANN model in the training cohort (Figure 4D). After 43,703 steps, the accuracy was 1.00 (95% CI, 0.92–1.00), and the sensitivity and specificity were 1.00 and 1.00, respectively (Table 1). Then, the parameters of the ANN model that passed in the training cohort were applied in the internal validation cohort and external validation cohort. The results showed that the accuracy, sensitivity, specificity and AUC of the model were 0.78 (95% CI, 0.62–0.94), 1.00, 0.61, and 0.800, respectively, in the internal validation cohort (Table 1 and Figure 5I). In the external validation cohort, the accuracy, sensitivity, specificity, and AUC were 0.90 (95% CI, 0.76–0.97), 1.00, 0.80, and 0.900, respectively (Table 1 and Figure 5J).

### Evaluation of different machine learning models and the selection of the most suitable model

**FIGURE 6:**
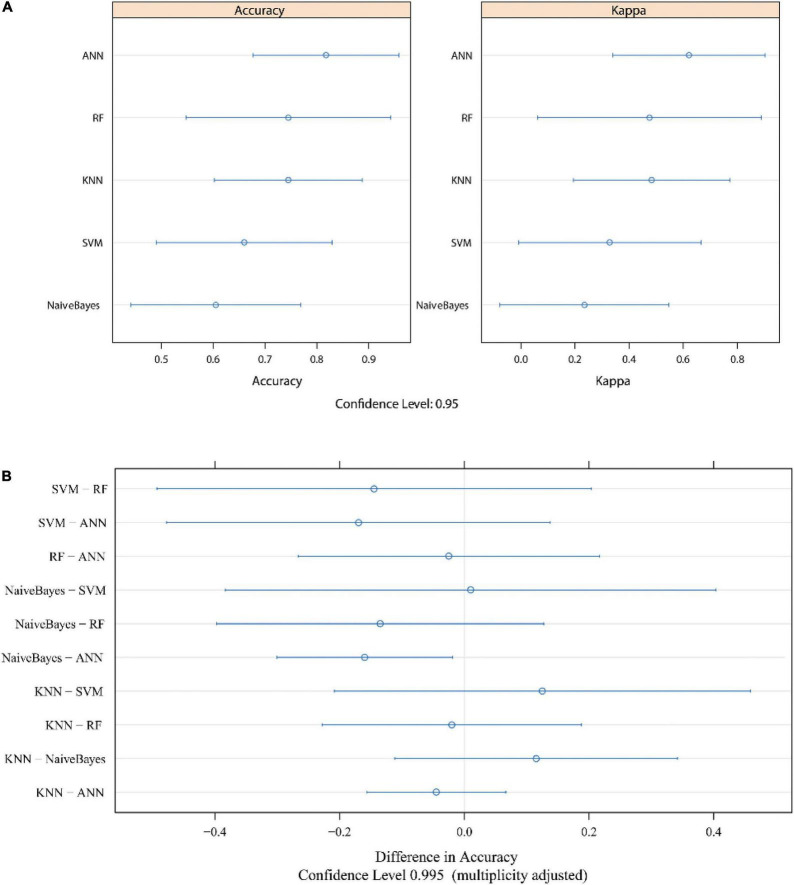
The evaluation of each model after cross validation. The accuracy and Kappa value are shown in panel **(A)**. Bonferroni’s test results are shown in panel **(B)**. The median numbers are represented by dots, and lines indicate the confidence level. ROC, receiver operating characteristic; AUC, area under the curve; KNN, k-nearest neighbor; SVM, support vector machine; RF, random forest; ANN, artificial neural network.

We evaluated the different machine learning models with the “resamples” function in R software after cross validation. The 95% CIs of the accuracy and kappa values after cross validation in each model are visualized in Figure 6A. Paired comparisons of the different models showed that the accuracy was significantly different between ANN and NB (Bonferroni’s test, *p* < 0.05) (Figure 6B). Although there was no significant difference between any of the other machine learning models (*p* > 0.05), we considered ANN to be the most suitable model for ASD prediction because of the high accuracy, sensitivity, specificity, and AUC, especially in the external validation cohort.

## Discussion

The prevalence of ASD has risen from 2 to 4 in 1,000 population to around 1% in large-scale population surveys (26). In clinical practice, we find most children are diagnosed between 2 and 3 years old. Briefly, ASD is much more common than previously believed, yet clinicians are often still confused regarding the early identification of ASD and its pathological mechanisms (27). The selection of novel potential biomarkers is crucial for the early identification and early treatment of children with ASD.

Cuproptosis is a new form of programmed cell death that is unlike apoptosis, pyroptosis, necroptosis, and ferroptosis (28). We selected the expression profiles of crucial genes in the cuproptosis signaling pathway from the brains of ASD mice and the peripheral blood of humans with ASD. The results of screening with the Boruta algorithm indicated that FDX1, DLAT, LIAS, and ATP7B were crucial genes in the cuproptosis signaling pathway for ASD in the present data. The results showed that ANN was the most suitable machine learning model for ASD prediction based on cuproptosis-related genes for the present cohort. This is the first study investigating biomarkers of the cuproptosis signaling pathway for ASD through the use of a machine learning algorithm.

Over the last 3 years, the Boruta algorithm has been used in many fields for feature selection, and it has shown reliability and stability with different evaluation methods (29–31). We also used the Boruta algorithm for the screening of risk genes for ASD in the cuproptosis signaling pathway, and we found that FDX1, DLAT, LIAS, and ATP7B were risk genes. Next, we found that these four selected genes were also expressed in the human brain, mainly in the mitochondria and Golgi apparatus, based on Human Protein Atlas immunofluorescence analysis. Thus, these four risk genes were closely related to brain function and cellular metabolism.

A previous study found that zinc-copper rhythmicity was disrupted in children with ASD (32). However, the previous mechanism could not exactly explain ASD, which may be because the cuproptosis signaling pathway in the cell cycle was reported just this year. FDX1 was found to be involved in copper-dependent cell death and could rescue cells from death by regulating mitochondrial metabolism (33). In this study, the expression of FDX1 was decreased in mice and humans with ASD (Figure 2). In addition, we found that FDX1 was expressed in mitochondria. The abnormal expression of FDX1 in ASD could cause a decrease in the expression of Fe–S cluster proteins and inhibit steroidogenesis (34). Abnormal steroid hormone levels have been found to contribute to the likelihood of autism (35). FDX1 deletion could inhibit DLAT lipoylation (28).

DLAT was another crucial risk gene in the cuproptosis signaling pathway for ASD identified by Boruta analysis in the present study. DLAT was specifically related to depression and anxiety in a chronic mild stress rat model (36). 6-Phosphogluconate dehydrogenase mutation led to reduced RNA and increased ROS by DLAT regulation (37). In addition, copper could induce the accumulation of DLA and activate the mitochondrial tricarboxylic acid cycle (8), which is consistent with our finding in the present study that DLAT is located in mitochondria. Thus, further studies should closely focus on the regulation of FDX1 and DLAT for mitochondrial function in ASD.

Lipoic acid synthetase is a protein target of lipoylation, and LIAS mutation has been described as being related to a defect in mitochondrial energy metabolism (38). In the present study, we found that LIAS expression was increased and was located in both the nucleoplasm and mitochondria. Previous studies found that mutations in LIAS were associated with non-ketotic hyperglycinaemia-like early-onset convulsions and encephalopathy combined with a defect in mitochondrial energy metabolism, and LIAS overexpression inhibited oxidative stress and inflammation (38–40). Therefore, we deduce that the accumulation of LIAS is not only related to Fe-S cluster synthesis and copper circulation but also indicates that oxidative stress levels may be increased in ASD patients.

The brain expression level of ATP7B was lower than that of other crucial genes based on the Human Protein Atlas; ATP7B plays an essential role in human physiology in the brain and liver. The deletion of ATP7B in cells and animals could decrease copper toxicity in Wilson’s disease (41). Copper homeostasis has been found to be associated with Alzheimer’s disease and Parkinson’s disease (42, 43). However, no study has revealed the role of this crucial regulatory gene in the copper concentration in ASD patients, and we hypothesize that ATP7B is another promising target for ASD research.

Although four crucial genes in the cuproptosis signaling pathway were screened, their power to predict ASD in suspected patients still needs to be investigated.

We next employed five machine learning methods for testing. The results showed that the accuracy of KNN, SVM, and RF was approximately 70% and up to 90% with the ANN model in the external validation cohort. Previous studies also show SVM, KNN, and RF have a decent prediction value for ASD (44, 45). While those studies are not verified in external validation cohorts, it is crucial to test the performance of prediction models in external validation cohorts. In the present study, each model was trained in the training cohort and validated in the internal cohort and external cohort.

However, the NB model showed poor overall performance and significantly poorer performance than the ANN model (*p* < 0.05). Some other studies have also found that the performance of NB was poor in comparison to other methods (24, 46). Additionally, NB’s poor performance might have been caused by the limited number of samples in the present study. Therefore, the NB method was not suitable for the present study.

The accuracies of KNN, SVM, and RF for ASD prediction did not differ much in the present study. In addition, sensitivity and specificity were also similar in the KNN, SVM, and RF models in the external validation cohort. Thus, KNN, SVM, and RF with selected genes in the cuproptosis signaling pathway have a similar ability to predict ASD in suspected patients.

Artificial neural network (ANN) was identified as the most suitable method for ASD prediction in the present study. For developing the DrugMiner web tool, Jamali et al. found that ANN outperformed NB, KNN, RF, and SVM (47). In addition, in reviews of machine learning methods, the authors also indicated that ANNs will be the dominant method in the field of biomedical science (24, 48).

Thus, detecting the expression levels of FDX1, DLAT, LIAS, and ATP7B in blood could predict the risk of ASD with ANN. These four risk factor genes could also be developed as microarrays for clinical examination. Future basic and experimental studies could also investigate the underlying pathophysiological mechanisms of the risk genes for ASD screened in the present study.

Furthermore, there are some limitations to the present study. The current study has a limited number of samples. The results need to be validated in a large sample size. Additionally, a prospective cohort study would be needed to detect the conclusions. However, we provided reliable machine learning methods, and four genes in the cuproptosis pathway that may be crucial for identifying mechanisms in autistic children.

## Conclusion

In the present study, on the basis of the results of screening with the Boruta algorithm, we selected FDX1, DLAT, LIAS, and ATP7B as crucial genes in the cuproptosis signaling pathway for ASD. The crucial risk genes were expressed in the brains of not only mice but also humans. ANN was the most suitable model for ASD prediction in the present study. We first reported that biomarkers in the cuproptosis-related signaling pathway had good power to predict ASD in suspected patients through different machine learning methods, which indicated that the cuproptosis signaling pathway may play a crucial role in ASD. The findings of the present study could contribute to the early identification of ASD in children and provide novel inspirations for investigations of the causes and treatments of ASD.

## Data availability statement

Publicly available datasets were analyzed in this study. This data can be found here: https://www.ncbi.nlm.nih.gov/gds with the accession numbers GSE72149, GSE81501, and GSE26415.

## Author contributions

YZ: conceptualization, methodology, software, data curation, and writing—original draft preparation. JG: visualization, investigation, supervision, software, validation, writing—reviewing and editing, and required funding. Both authors read and approved the manuscript.

## Footnotes

1
https://www.proteinatlas.org
